# Promoter Methylation Status of Survival-Related Genes in MOLT- 4 Cells Co-Cultured with Bone Marrow Mesenchymal Stem Cells under Hypoxic Conditions

**DOI:** 10.22074/cellj.2018.5101

**Published:** 2018-03-18

**Authors:** Milad Ahani-Nahayati, Saeed Solali, Karim Shams Asenjan, Ali Akbar Movassaghpour Akbari, Mehdi Talebi, Milad Zadi Heydarabad, Sina Baharaghdam, Majid Farshdousti Hagh

**Affiliations:** 1 Immunology Research Center, Tabriz University of Medical Sciences, Tabriz, Iran; 2 Drug Applied Research Center (DARC), Tabriz University of Medical Sciences, Tabriz, Iran; 3 Hematology and Oncology Research Center, Tabriz University of Medical Sciences, Tabriz, Iran

**Keywords:** Acute Lymphoblastic Leukemia, DNA Methylation, Mesenchymal Stem Cell, Promoter Regions

## Abstract

**Objective:**

DNA methylation is a well-studied epigenetic mechanism that is a potent arm of the gene expression controlling machinery. Since the hypoxic situation and the various cells of bone marrow microenvironment, e.g. mesenchymal stem cells, play a role in the in vivo and in vitro biology of leukemic cells, we decided to study the effects of hypoxia and mesenchymal stem cells (MSCs) on the promoter methylation pattern of *BAX* and *BCL2* genes.

**Materials and Methods:**

In this experimental study, the co-culture of MOLT-4 cells with MSCs and treatment with CoCl_2_ was done during 6, 12, and 24 hour periods. Total DNA was extracted using commercial DNA extraction kits, and sodium bisulfite (SBS) treatment was performed on the extracted DNA. Methylation specific polymerase chain reaction (MSP) was used to evaluate the methylation status of the selected genes’ promoter regions.

**Results:**

The *BAX* and *BCL2* promoters of untreated MOLT-4 cells were in partial methylated and fully unmethylated states, respectively. After incubating the cancer cells with CoCl_2_and MSCs, the MSP results after 6, 12, and 24 hours were the same as untreated MOLT-4 cells. In other words, the exposure of MOLT-4 cells to the hypoxia-mimicry agent and MSCs in various modes and different time frames showed that these factors have exerted no change on the methylation signature of the studied fragments from the promoter region of the mentioned genes.

**Conclusion:**

Hypoxia and MSCs actually have no notable effect on the methylation status of the promoters of *BAX* and *BCL2* in the specifically studied regions. DNA methylation is probably not the main process by which MSCs and CoCl_2_ induced hypoxia regulate the expression of these genes. Finally, we are still far from discovering the exact functional mechanisms of gene expression directors, but these investigations can provide new insights into this field for upcoming studies.

## Introduction

One of the most serious problems affecting children is acute lymphoblastic leukemia (ALL) that mainly occurs in the first 2 to 5 years of life. ALL is associated with invasive properties and uncomfortable and life- threatening complications, still, it is one of the most curable malignancies of young age (1, 2). Because of the unique situation of the bone marrow cavity, these malignant cells survive a special condition compared to the cells of other organs. There are mesenchymal stem cell (MSC) in the bone marrow microenvironment, which act on hematopoietic elements, as well as tumor cells, supported through different ways (3, 4). 

There are some studies that focus on growth inhibition and limiting the proliferation effects of MSCs on leukemic cells. In total, MSCs are said to be either the helpers or the enemies of malignant cells (5). On the other hand, the bone marrow cavity has a hypoxic condition and its low oxygen tension provides a special situation for hematopoietic cells and other components of hematopoiesis, which clearly affects the status of transcription factors and gene expression profiles of the cells (6). One of the essential effects of hypoxia is the induction of hypoxia inducible factor-1 (HIF1) expression, a transcription factor that is the cause of various events including angiogenesis, increase in metabolism, and the initiation of the production of diverse proteins. This case is more considerable for the bone marrow resident cells (7). To mimic the hypoxic situation, we used CoCl_2_ as a HIF1 inducer (8). 

The most crucial genes involved in cell survival and death are the *BCL2* family members, among which, *BAX* and *BCL2* have great importance. Dysregulation of *BAX* and *BCL2* expression is evident in various types of cancers (9). For example, a common feature of cancerous cells is overexpression of the *BCL2* that is frequently linked with poor prognosis and chemotherapy resistance (10). Epigenetics is one of the central mechanisms for such changes in mammalian cells and DNA methylation is the most well-known epigenetic mechanism involved in the regulation of gene expression. Methylation mainly occurs at specific positions in gene promoters called CpG islands, in which a methyl group attaches to the 5^th^ atom of the ring in the cytosine base in a non-exclusive manner and inhibit transcription factor adherence to the DNA, and consequently cause down- regulation of gene expression (11-13).

According to the "two hit hypothesis", the aberrant methylation can be considered as a third factor in carcinogenesis (13). Nowadays, epigenetic mechanisms are interesting targets for cancer treatment studies. Of course, these therapeutic strategies are in their infancy and they sometimes show incompatible consequences (14), but the imagination of a positive future for them is not too implausible (15).

Since hypoxia can provide a connection between the extra-cellular environment, methylation of DNA, and carcinogenesis, in the current study, the methylation levels of *BAX* and *BCL2* genes, as the critical genes involved in cell death and survival, are evaluated via methylation specific polymerase chain reaction (MSP) in MOLT-4 cells, a T-ALL cell line, co-cultured with bone marrow MSCs under hypoxic condition. 

## Materials and Methods

In this experimental study that has been approved by the Ethics Committee of the Tabriz University of Medical Sciences, the MOLT-4 cells were provided from Pasteur Institute Cell Bank (Tehran, Iran) and cultured in RPMI 1640 (Gibco Laboratories, Grand Island, NY) medium containing 10% fetal bovine serum (FBS, Gibco Laboratories, Grand Island, NY). The cells were incubated in 5% CO_2_ incubator at 37°C. Bone marrow MSCs were purchased from Royesh Stem Cell Biotechnology Institute Cell Bank (Tehran, Iran) and cultured in DMEM (Dulbecco’s Modified Eagle Medium, Gibco Laboratories, Grand Island, NY) including 10% FBS, then incubated in 5% CO_2_ at 37°C humidified atmosphere.

### Evaluation of direct CoCl_2_ toxicity for the cells

The cytotoxicity of CoCl_2_ was measured by Trypan Blue and 3-(4,5-dimethyl thiazol-2-yl)-5-diphenyl tetrazolium bromide (MTT) assays to reach the maximum levels of HIF1 induction with no significant cell death. The cytotoxicity of CoCl_2_ on the MOLT-4 cells was assessed using treatment of the cells with differing concentrations of cobalt chloride with various timings (25, 50, 100, 150, and 200 µM CoCl_2_).

### MOLT-4 cell co-culture with mesenchymal stem cells and hypoxic treatment

The MSCs were seeded at a density of 5×10^4^ cells/cm^2^ in plates containing DMEM. Once every three days, the medium was replaced with fresh medium, until the MSC feeder layer reached confluence (70-80%). Next, 2×10^6^ MOLT-4 cells were added to the culture and incubated for 6, 12, and 24 hours. Following that, we treated the MOLT- 4 and MSCs, in various designed modes, with 100 µM CoCl_2_. The cells were incubated in a 5% CO_2_ incubator at 37°C for 6, 12, and 24 hours.

### Extraction of DNAand treatment with sodium bisulfite

Extraction of total DNA from MOLT-4 cells was done at 6, 12, and 24 hours after the co-culture using a DNA extraction Kit (YT9040) according to the manufacturer’s directions. The DNA quality was evaluated by spectrophotometry and calculated by the ratio of the optical density of DNA (260 nm) and optical density of protein (280 nm). Sodium bisulfite (SBS) treatment was applied to the extracted DNA. It transforms unmethylated cytosines to uracils. 

Freshly prepared solutions of SBS and hydroquinone were utilized. Primary denaturation of DNA was initiated with 2 M solution of NaOH, then the tube was incubated for 20 minutes at 37°C. The treatment of denaturated DNA with 3 M SBS (pH=5) and 10 mM hydroquinone was performed, then covered under a layer of mineral oil and incubated for 16 hours at 50°C.

At this point, the purification of modified DNA was performed using YT9040 DNA purification columns as stated by the manufacturer, then eluted into 150 µl of elution buffer. Subsequently, desulfonation was accomplished by adding 3 M NaOH solution and incubating at room temperature for 5 minutes. The neutralization of the solution was done by adding 3M ammonium acetate (pH=7.0). Next, by adding 4 times the current volume in ethanol, the DNA was precipitated. Drying and resuspending in 20 µl double distilled water were the following steps. SBS treated DNA was used directly for MSP or stored at -20°C.

### Genomic DNA methylation using SssI methylase enzyme

Methylated DNA needed to be prepared as the positive control for MSP. DNA was extracted from peripheral blood and methylated with the Sss1 methylase enzyme (Biolabs, New England, US) following producer’s directions (16). The methylated DNA was immediately extracted via YT9040 DNA extraction kit and exposed to SBS treatment, then used as a positive control for the methylated DNA-specific primer (meth primer). 

### Methylation specific polymerase chain reaction

MSP was performed to evaluate the methylation status of the *BAX* and *BCL2* promoters. MSP-specific primers are able to discern between methylated and unmethylated DNA sequences. The designing of MSP primers was done through MethPrimer software (17). The forward and reverse primers for *BAX* and *BCL2* genes have been listed (Table 1). 

For the MSP test, the bisulfite-treated DNA of MOLT-4 cells was used along with control samples including SssItreated DNA, meth primer positive control, peripheral blood SBS-treated DNA, a positive control for unmethylated DNA-specific primer (unmeth primer), normal human DNA with no SBS treatment as the negative control for both primers, and No-DNA sample as a negative control for the PCR reaction. The PCR mixture contained 12.5 µL of PCR master mix (Ampliqon, DENMARK), 1.25 µL of each forward and reverse primers (in a final concentration of 0.5 pM), bisulfite-modified DNA (150 ng), and double distilled water, in the final volume of 25 µl. The thermal cycler (MyCycler-BioRad) was used for amplification. The time and thermal periods of PCR were as follows: 5 minutes at 95°C for 1 cycle, subsequently 45 seconds at 95°C, 30 seconds at 59°C, then 45 seconds at 72°C, for 35 cycles. Next, an ultimate extension for 5 minutes at 72°C. Finally, in order to separate the PCR reaction products, electrophoresis was carried out on a 1.5% agarose gel and colored using safe stain, then studied under UV light.

### Statistical analysis 

Three separate tests have been performed for each value and the data reported as mean ± SD. The significance of data has been presented as P<0.05 by the t student test using SPSS 16 (SPSS Inc., Chicago, IL, USA).

## Results

### Cell toxicity assessment of CoCl_2_-treated cells

**Fig.1:**
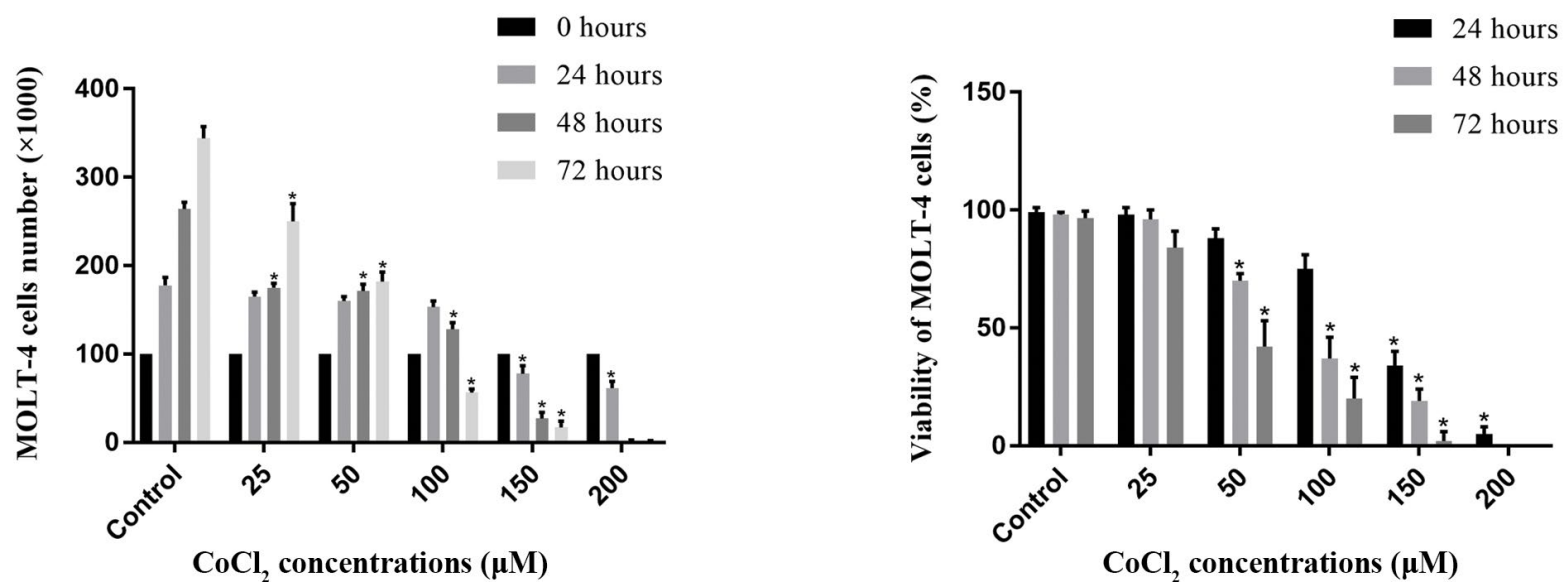
The evaluation of direct cytotoxicity of CoCl_2_ for the MOLT-4 cells. The MOLT-4 cells have been treated with different concentrations of COCl_2_ (25, 50, 100, 150, 200 µM) during 24, 48, and 72 hours. A. MOLT-4 cells counting using Trypan blue and B. MOLT-4 cells viability assessment using MTT assay. The data shows that the highest possible concentration of CoCl_2_ for HIF1 induction without significant cell death is 100 µM in 24 hours. Error bars represent standard deviations and data significance levels are shown as *P<0.05.

CoCl_2_ is a hypoxia mimicry agent that induces HIF1 expression in a dose-dependent manner. Our results showed that CoCl_2_ in less than 100 µM doses, doesn’t significantly suppress cell growth in 24, 48, or 72 hours, and in more than 100 µM doses, CoCl_2_ was lethal to the cells in every exposure time. Therefore, 100 µM concentration of CoCl_2_ was chosen as the best dose during 24 hours. Indeed, this step has been conducted to achieve the highest possible dose of CoCl_2_, to reach the maximum possible amount of HIF1, in which the cells can stay alive (Fig.1).

**Table 1:** The MSP-specific primers of BAX and BCL2 genes


Target sequence	Primer sequencing (5ʹ- 3ʹ)	Tₐ^*^ (C˚)	Amplicon size (bp)	Amplified region^**^

BAX	Meth-F: GTATTAGAGTTGCGATTGGACGG	59	162	48954657-48954819
	Meth-R: AAAATAACCGCTACCCCGC			
	Unmeth-F: GAAGGTATTAGAGTTGTGATTGGATG	58.5	167	48954653-48954820
	Unmeth-R: CAAAATAACCACTACCCCACAA			
BCL2	Meth-F: GTTTTTAGCGTTCGGTATCGG	60	192	63319549-63319741
	Meth-R: AAATCTCTATCCACGAAACCGC			
	Unmeth-F: GGGTTTTTAGTGTTTGGTATTGG	59	194	63319549-63319743
	Unmeth-R: AAATCTCTATCCACAAAACCACTTC			


*; Annealing temperature and **; Nucleotide numbers.

### Analysis of MOLT-4 cells growth curve in co- culture with mesenchymal stem cells under hypoxic conditions 

**Fig.2:**
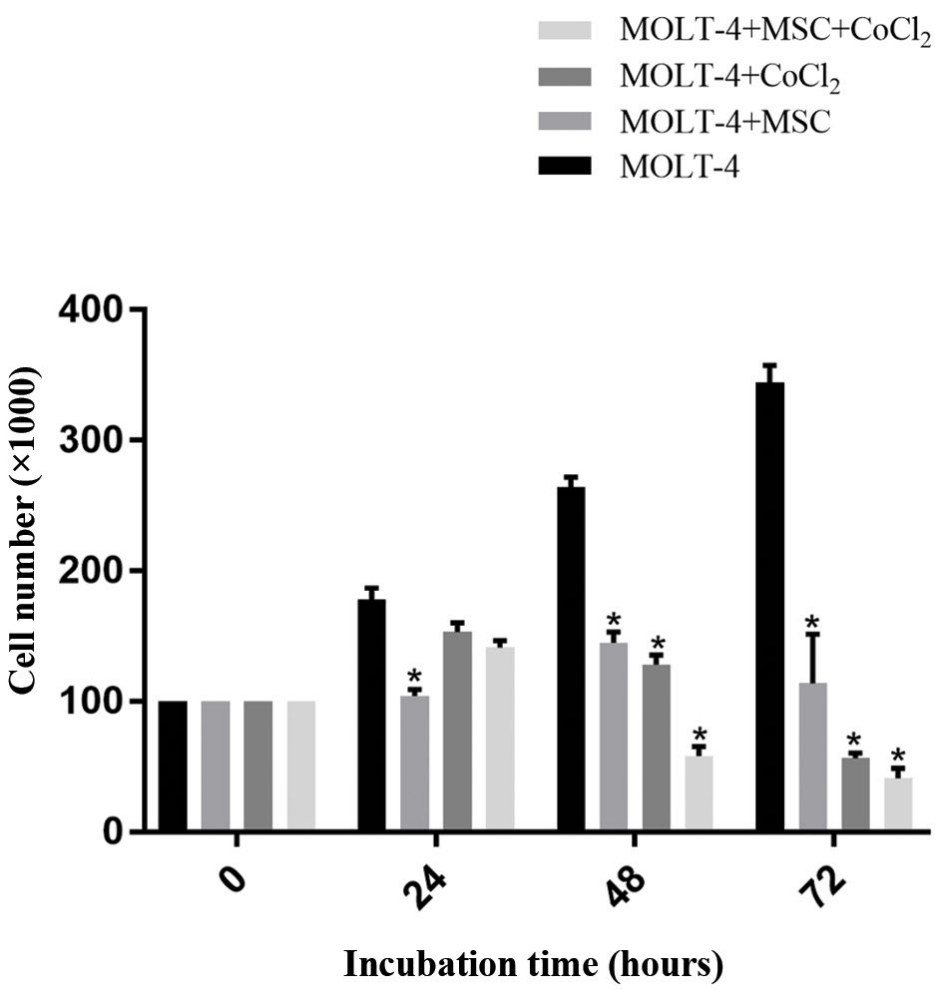
MOLT-4 cells counting under different conditions of cell culture. Cell counting was done after 24, 48, and 72 hours. Error bars represent standard deviations and data significance levels are shown as *P<0.05.

Untreated and cobalt-exposed (100 µM, 24, 48, and 72 hours) MOLT-4 cells were cultured in matching numbers. After 24, 48, and 72 hours, the cells were counted using Trypan blue staining. Diverse conditions were provided for MOLT-4 cells in the culture medium (besides MSCs, CoCl_2_, MSCs and CoCl_2_). Our outcomes revealed that MSCs have suppressed the growth of MOLT-4 cells in every timeframe. In addition, hypoxia alone also has an inhibitory effect on MOLT-4 cells proliferation in all time periods. As expected, when we exposed MOLT-4 cells to MSCs and hypoxia simultaneously, the cells growth showed another drop (Fig.2). 

### The methylation status of the *BAX* gene promoter 

**Fig.3:**
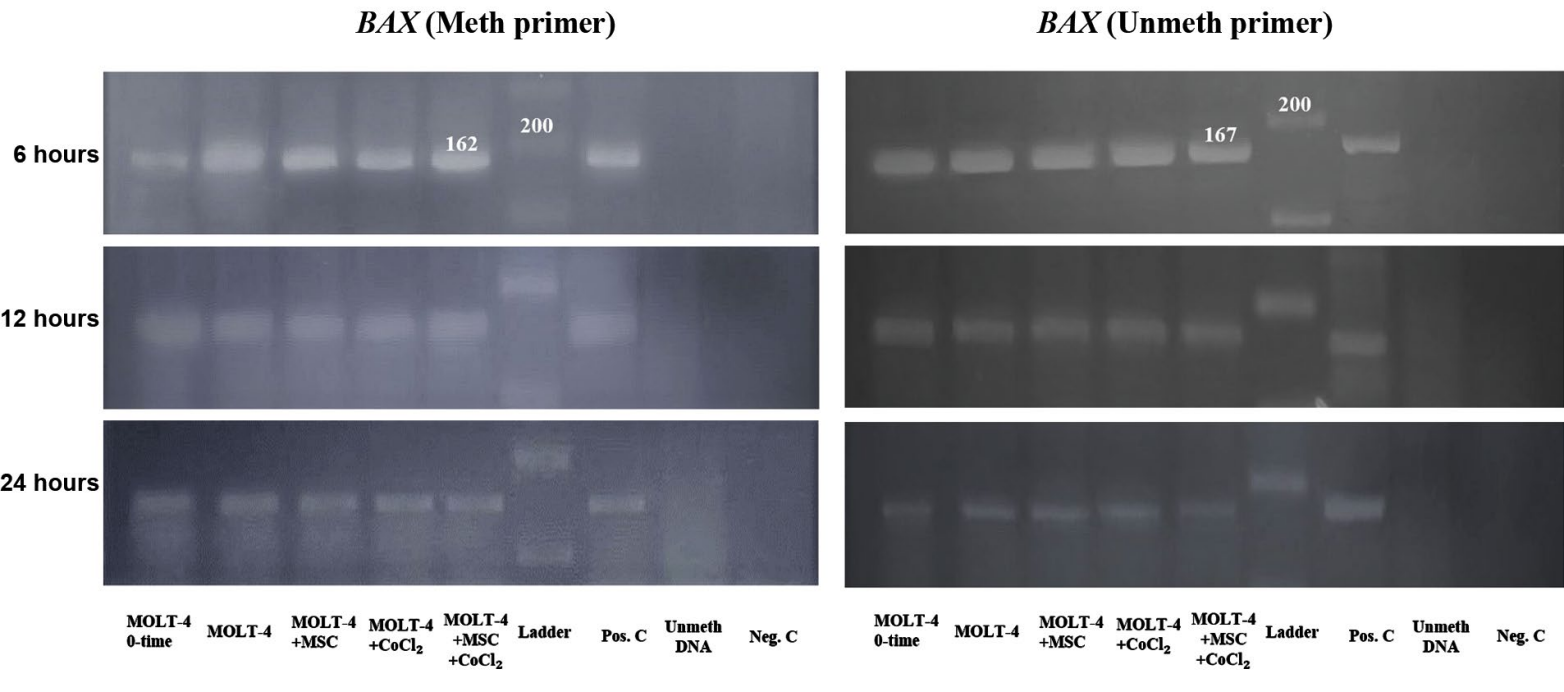
The MSP results for the BAX gene promoter in various conditions at 6, 12, and 24 hours with Meth and unmeth primers. The condition of each line has been described in the bottom of the picture. The positive result with the meth primer generates a 162bp product, and with the unmeth primer, it generates a 167 bp product. As shown in the picture, the studied sequence of the BAX gene promoter revealed positive results with both meth and unmeth primers, indicating its partial methylation status in all conditions.

Investigating the *BAX* gene promoter methylation status in the untreated MOLT-4 cells showed that both meth and unmeth primers made positive results in the MSP test. These results were constant during all of the study periods and none of them showed absolute methylation or unmethylation for this gene’s promoter. The final results of the MSP test were positive for meth and unmeth primers of *BAX* gene in the various designed conditions at 6, 12, and 24 hours (Fig.3). 

### The methylation status of the *BCL2* gene promoter

**Fig.4:**
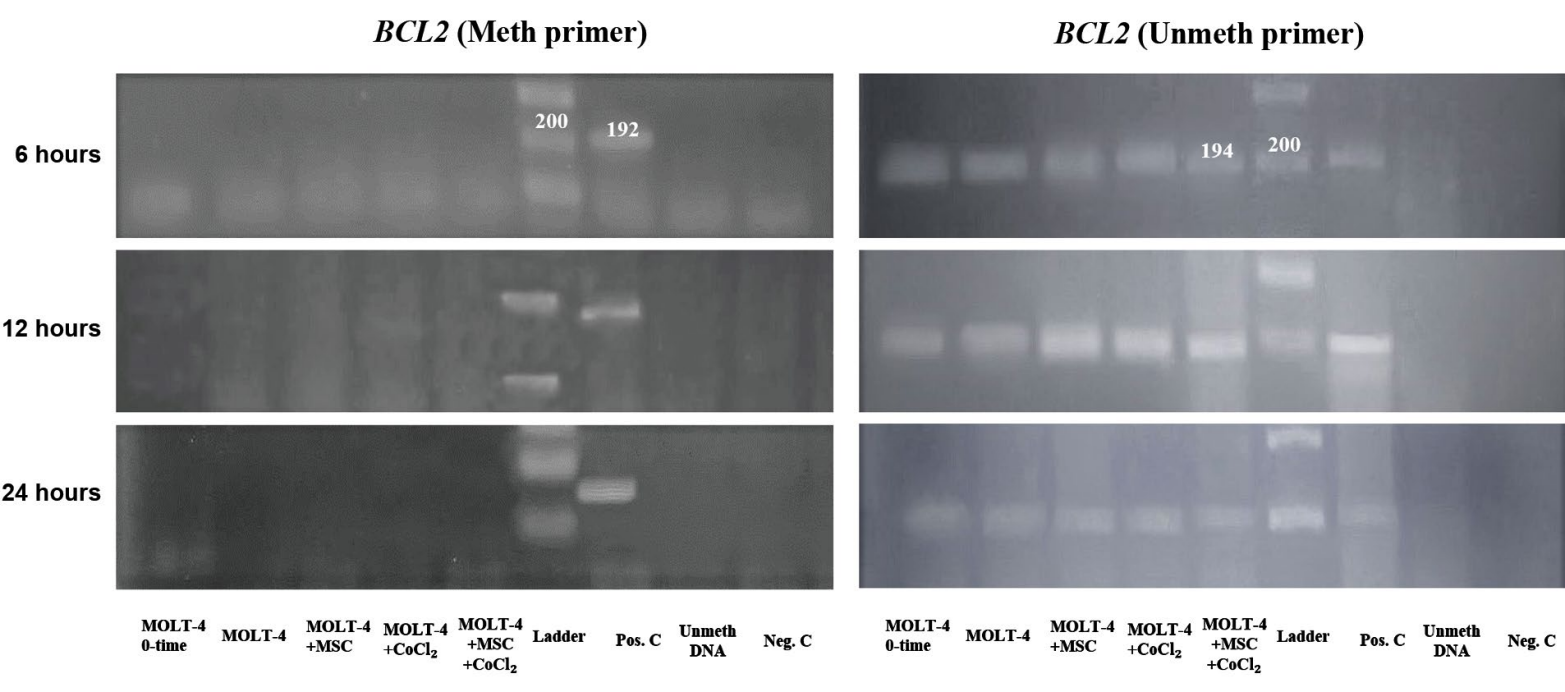
The MSP results for the *BCL2* gene promoter in various conditions at 6, 12, and 24 hours with meth and unmeth primers. The condition of each line has been described in the bottom of the picture. The positive result with the eth primer generates a 192 bp product, and with unmeth primer, it generates a 194bp product. As shown in the picture, the studied sequence of *BCL2* gene promoter revealed negative results with the meth primer, and positive results with the unmeth primer, indicating its fully-unmethylated status under all conditions.

The MSP results showed that the promoter of *BCL2* in the untreated MOLT-4 cells was in the unmethylated state. As shown in Figure 4, the meth primer for BCL2, in contrast to the unmeth primer, had a negative result and showed no visible band after electrophoresis. The BCL2 gene remained in the unmethylated status in all various conditions including MOLT-4 cells, MOLT-4 and MSCs, MOLT-4 and CoCl_2_, MOLT-4 and MSCs with CoCl_2_ (Fig.4).

## Discussion

HIF1 is a key regulator of cell response to hypoxia that can impress several mechanisms in the cell and have a critical role in carcinogenesis (18). It also has an effect on epigenetic mechanisms including DNA methylation. Liu et al. (9) reported that HIF1 expression can result in several genes being demethylated through a decrease in the steady-state form of S-adenosyl methionine (SAM). As expected, the anti-apoptotic genes of cancerous cells routinely have high expression levels (19). *BCL2* is one of the fundamental anti-apoptotic genes that is important for the unnatural survival of malignant cells and their resistance to chemotherapy (20). Since the methylation of the promoter region can silence a gene, if promoter loses its methylation, the gene can reach to higher levels of expression (21).

Our results have shown that the *BCL2* gene promoter in untreated MOLT-4 cells was in the fully unmethylated state, similar to Chatterton et al.’s investigations on ALL cells in 2014 (22). In the following steps, to make the environment of the MOLT-4 cells closer to the bone marrow microenvironment, we co-cultured them with MSCs that are the essential components of the BM stroma, and treated them with CoCl_2_, as a HIF1 inducing factor. Of course, CoCl_2_ has some limitations like its toxicity, nonetheless, because of some of its benefits, like accessibility, it is a commonly used substance in numerous studies (23-25).

The aforementioned conditions for MOLT-4 cells in the present study weren’t able to affect the methylation status of the *BCL2* promoter. Another study by our team, investigating *BAX* and *BCL2* expression levels in similar situations through the real-time PCR, showed that *BCL2* expression in the untreated MOLT-4 cells was higher than normal T cells, and co-culturing with MSCs and treatment with CoCl_2_ have increased this expression even more (26). This significant increase in *BCL2* expression is in contrast to some studies like Wang et al. (13), but in complete coordination with the results of the current study. The correlation between the increased expression of *BCL2* and loss of methylation is totally logical. Regarding the *BCL2* functions, we can state that the MSCs and hypoxic condition have a favorable effect on this gene’s expression and it has no association with promoter methylation. So, other mechanisms might be involved in this increase (27, 28).

The investigations about the *BCL2* gene expression and its promoter methylation in various cancers have led to different and sometimes conflicting results, such as both up and down regulation of this gene by various mechanisms (13, 29, 30). It is stated by Hogarth and Hall (29) that the *BCL2* expression levels can’t be useful in the prognosis of the ALL patients, but obviously, the downregulation of this gene, which might be happening via the promoter methylation processes, can be a valuable factor in tumor regression. On the other hand, MSCs can secrete several hematopoiesis-supporting and niche enhancing cytokines under hypoxia (3). Frolova et al. (31) provided similar conditions for ALL cancer cells and demonstrated diminished apoptosis in these cells. It is clear that the *BCL2* expression increase is supportive for malignant MOLT-4 cells, but our data shows that this case has no association with the methylation machinery.

The *BAX* gene is a central pro-apoptotic gene that can cause cell death and limit cancer progression (32). The *BAX* gene promoter was methylated in many different tumors, according to several reports (33-35). Our results have shown that this gene was in the partial methylation state in the untreated MOLT-4 cells. It means that both meth and unmeth primers for the *BAX* gene promoter exhibited positive results in MSP test.

Our findings showed no change in the promoter methylation levels of the *BAX* gene, which was in the partial methylated mode before and after the co-culture with MSCs and treatment with CoCl_2_. Of course, MSP is a non-quantitative method and is not capable of determining the precise percentage of methylation (36), thus, the difference between various results of partial methylation wasn’t measurable for us. However, the evaluation of *BAX* expression showed that the gene expression had no remarkable changes before and after co-culture with MSCs and treatment with CoCl_2_ (26). 

The *BAX* gene only showed an insignificant increase in the various conditions of the study compared with the untreated MOLT-4 cells and it is actually explainable with our results from MSP. In fact, the loss of substantial changes in gene expression is in line with the insignificant alterations in the gene promoter methylation levels. So, even if methylation has a role in *BAX* expression process of MOLT4 cells, we cannot prove this point with the current outcomes. In total, as the MSCs and hypoxia don’t apply any significant impact on *BAX* expression, they don’t markedly change the methylation levels. Just like the inconsistencies about *BCL2* methylation and expression status in ALL cells, there are many unlike information about *BAX* gene too. For example, Wang et al. (13) published article mentioned that the *BAX* gene is increased in ALL cells, which was supported by Kaparou et al. (37) studies, whereas Prokop et al. (38) have reached a completely opposite conclusion.

Although the increase of *BAX* expression was insignificant, it might be due to the demethylation of its promoter region. It should be assessed by more accurate methods than MSP, e.g. Bisulfite-Sequencing PCR (BSP) and Methylation-Sensitive-High Resolution Melting (MS-HRM) (39, 40). Of course, the methylation is a time- consuming process and should be assessed in longer time periods. We know it is possible that our results might vary after more time, and further investigations. 

## Conclusion

Although limited, our study attempts to bring new parameters together, which were rarely addressed before, and examine their effects on survival-related genes of a malignant human cell line. We showed that MSCs and hypoxia couldn’t change the methylation signature of *BAX* and *BCL2* promoters in particular studied regions. Obviously, the promoter methylation levels may undergo different alterations in other regions that weren’t considered in the current study. This material can broaden our perspective about the mechanisms involved in the regulation of cancer cell survival, and someday, may be helpful for novel therapeutic strategies. 
